# Signal mining of adverse reactions for neuraminidase inhibitors in pregnant women: a disproportionality analysis based on the FDA adverse event reporting system database

**DOI:** 10.3389/fmed.2026.1821866

**Published:** 2026-06-16

**Authors:** Bo Cao, Mingfeng Shen, Chenchen Du, Qiuping Song, Zhongjie Ma

**Affiliations:** 1 Department of Pharmacy, Suzhou Ninth People's Hospital, Suzhou Ninth Hospital Affiliated to Soochow University, Suzhou, Jiangsu, China; 2 Department of Rehabilitation, Suzhou Ninth People’s Hospital, Suzhou Ninth Hospital Affiliated to Soochow University, Suzhou, Jiangsu, China

**Keywords:** disproportionality analysis, FAERS, neuraminidase inhibitors, pharmacovigilance, pregnancy

## Abstract

**Background:**

This study aimed to comprehensively analyze the adverse events associated with neuraminidase inhibitor (NAI) use in pregnant women in real-world clinical settings, providing a valuable reference for the management of anti-influenza treatment in this population.

**Methods:**

The study extracted data from the Food and Drug Administration (FDA) Adverse Event Reporting System (FAERS) database, spanning from the first quarter of 2004 to the third quarter of 2025. Signal detection was performed using the reporting odds ratio (ROR) and Bayesian confidence propagation neural network (BCPNN) methods. Adverse events (AEs) were classified according to the system organ class (SOC) in the Medical Dictionary for Regulatory Activities (MedDRA) version 27.0.

**Results:**

From Q1 2004 to Q3 2025, a total of 3,687 ADE reports were retrieved in this study, including 3,211 reports for oseltamivir, 471 reports for zanamivir, and 5 reports for peramivir. For oseltamivir, significant AEs at SOCs were injury, poisoning, and procedural complications; pregnancy, puerperium, and perinatal conditions; and general disorders and administration site conditions. The five strongest AE signals were proctitis, infantile acne, congenital intestinal obstruction, no adverse event, and placental necrosis. For zanamivir, significant AEs at SOCs were injury, poisoning, and procedural complications; pregnancy, puerperium, and perinatal conditions; and respiratory, thoracic, and mediastinal disorders. The five strongest AE signals were delivery, bronchospasm, pneumothorax, exposure during pregnancy, and product quality issue.

**Conclusion:**

The study findings align with current guidelines recommending neuraminidase inhibitors as the primary antiviral treatment of pregnant women with influenza. Our study may offer valuable evidence to support the clinical use of neuraminidase inhibitors, particularly in identifying adverse events and ensuring their safe administration in women during pregnancy.

## 1 Introduction

Influenza is an acute infectious disease that threatens pregnant mothers with an increased risk of complications and worse clinical outcomes (1, 2). During seasonal influenza or pandemic influenza outbreaks, women during pregnancy are more susceptible to severe infection due to normal changes in immunology, physiology, and anatomy during pregnancy (3). It has been reported that maternal influenza virus infection is associated with adverse birth outcomes (4, 5), suggesting that influenza should be prevented and treated in this population.

The World Health Organization and its many guidelines have recommended neuraminidase inhibitors (NAIs) for the treatment of pregnant women with influenza (6–8), including oral oseltamivir, inhaled zanamivir, and intravenous peramivir. Previous studies have demonstrated that neuraminidase inhibitor use during pregnancy did not increase the overall risk of adverse maternal or fetal outcomes (9–11).

Pharmacovigilance studies play an important role in supplementing real-world drug use safety (12). The US Food and Drug Administration Adverse Event Reporting System (FAERS) is a public database established to facilitate post-market safety monitoring of drugs and therapeutic products by the FDA (13, 14). It serves as a valuable resource for the early detection and identification of potential adverse effects, helping to identify safety issues related to drug therapy in the real world based on a large population and to provide references and guidance for drug regulation in clinical practice (15). To the best of our knowledge, there are no comprehensive pharmacovigilance analyses of NAI use among pregnant women based on FAERS data. Therefore, the objective of this study was to identify adverse drug event (ADE) signals associated with NAI use among pregnant women by using disproportionality analysis, providing evidence-based insights that can guide clinical practices and inform regulatory decisions regarding the use of NAIs among pregnant women.

## 2 Materials and methods

### 2.1 Data sources and processing

**Figure 1:**
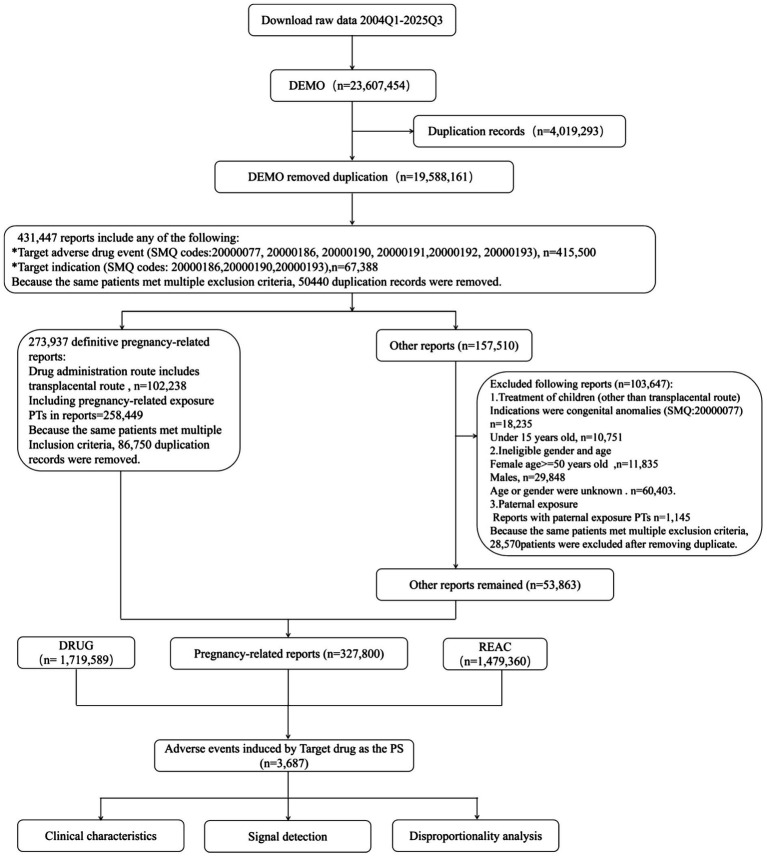
The flow diagram for selecting neuraminidase inhibitor-related AEs from the FAERS database.

The study sourced data from the FDA’s FAERS database, extracting adverse event reports related to neuraminidase inhibitor exposure during pregnancy from the first quarter of 2004 to the third quarter of 2025. The generic name of the target drug is limited to “OSELTAMIVIR,” “ZANAMIVIR” and “PERAMIVIR.” The American Standard Code for Information Interchange (ASCII)-encoded data packets were downloaded, and the data were processed using SAS 9.4. The FAERS database used the MedDRA 27.0 to match the preferred terms (PTs) for neuraminidase inhibitor adverse reactions and also listed the system organ classes (SOCs) that corresponded to these PTs. Only reports identifying neuraminidase inhibitors (oseltamivir, zanamivir, and peramivir) as the primary suspect (PS) associated with AEs were considered in this study. The flow diagram of data selection is illustrated in Figure 1.

### 2.2 Pregnancy-related reports retrieval

To identify pregnancy-related reports, the previously described method in FAERS was used in our study (16, 17)^.^ We used SMQ codes 20000077, 20000186, 20000190, 20000191, 20000192, and 20000193 in adverse drug event fields, with a total of 415,500 records. SMQ codes 20000186, 20000190, and 20000193 were used to extract terms related to pregnant mothers in the indication fields, and 67,388 records were included. Since the same patients met multiple criteria in both the adverse event and indication fields, 50,440 duplicates were removed, resulting in a total of 431,447 reports. Cases including transplacental administration and pregnancy-related exposure PTs were identified as definitive pregnancy-related reports. A total of 273,937 records were included after removing 86,750 duplicate reports. In addition, 103,647 records were excluded due to treatment of children, ineligible gender and age, and paternal exposure. Therefore, after processing, a total of 327,800 pregnancy-related records were included in our final analysis (Figure 1).

### 2.3 Statistical analysis

Based on the rationale of disproportionality analysis and Bayesian analysis, the reporting odds ratio (ROR) (18) and Bayesian Confidence Propagation Neural Network (BCPNN) (19) algorithms were applied to investigate the associations between the drug and the specified adverse events. The threshold is set as follows: a ≥ 3, the lower limit of the ROR 95% confidence interval is > 1, and the lower confidence interval of IC (IC-2SD) > 0. The larger the values of ROR and BCPNN were, the stronger AE signals were, indicating a stronger statistical relationship between the target drug and the target AEs. The equations and criteria for the two algorithms are shown in Table 1, and the disproportionality analysis through a 2 × 2 contingency table is shown in Supplementary Table S1.

**Table 1:** Summary of algorithms.

Algorithms	Equation^a^	Criteria
ROR	$\mathrm{ROR}=\frac{\left(a/c\right)}{\left(b/d\right)}=\frac{\mathrm{ad}}{\mathrm{bc}}$ $\mathrm{SE}(\text{InROR})=\sqrt{\left(\frac{1}{a}+\frac{1}{b}+\frac{1}{c}+\frac{1}{d}\right)}$ $95\%\mathrm{CI}=e^{\text{In}(\mathrm{ROR})\pm 1.96\;\sqrt{\left(\frac{1}{a}+\frac{1}{b}+\frac{1}{c}+\frac{1}{d}\right)}}$	a ≥ 3, ROR ≥ 1, 95% CI (lower limit) > 1
BCPNN	$\mathrm{IC}=\log_{2}\frac{p(x,y)}{p(x)p(y)}=\log_{2}\frac{a(a+b+c+d)}{\left(a+b)(a+c\right)}$ $E(\mathrm{IC})=\log_{2}\frac{\left(a+\gamma 11)(a+b+c+d+\alpha )(a+b+c+d+\beta \right)}{\left(a+b+c+d+\gamma )(a+b+\alpha 1)(a+c+\beta 1\right)}$ $V(\mathrm{IC})=\frac{1}{{\left(\ln2\right)}^{2}}\{[\begin{array}{l} \frac{(a+b+c+d)-a+\gamma -\gamma 11}{(a+\gamma 11)(1+a+b+c+d+\gamma }+\frac{(a+b+c+d)-(a+b)+\alpha -\alpha 1}{\left(a+b+\alpha 1)(1+a+b+c+d+\alpha \right)}\\ +\frac{(a+b+c+d)-(a+b)+\beta -\beta 1}{\left(a+c+\beta 1)(1+a+b+c+d+\beta \right)} \end{array}]\}$ $\gamma =\gamma 11\frac{\left(a+b+c+d+\alpha )\;(a+b+c+d+\beta \right)}{\left(a+b+\alpha 1)\;(a+c+\beta 1\right)}$ $\mathrm{IC}-2\mathrm{SD}=E(\mathrm{IC})-2\sqrt{V(\mathrm{IC})}$ α1=β1=1;α=β=2;γ11=1	No signal (−): IC-2SD ≤ 0 Weak signal (+): 0 < IC-2SD ≤ 1.5 Medium signal (++): 1.5 < IC-2SD ≤ 3 Strong signal (+++): IC-2SD > 3

a ROR, reporting odds ratio; a, number of reports containing both the suspect drug and the suspect adverse drug reaction; b, number of reports containing the suspect adverse drug reaction with other medications (except the drug of interest); c, number of reports containing the suspect drug with other adverse drug reactions (except the event of interest); d, number of reports containing other medications and other adverse drug reactions; CI, confidence interval; *χ*^2^, chi-square; BCPNN, Bayesian confidence propagation neural network; IC, information component; IC-2SD, the lower confidence interval of IC.

## 3 Results

### 3.1 General characteristics

**Figure 2:**
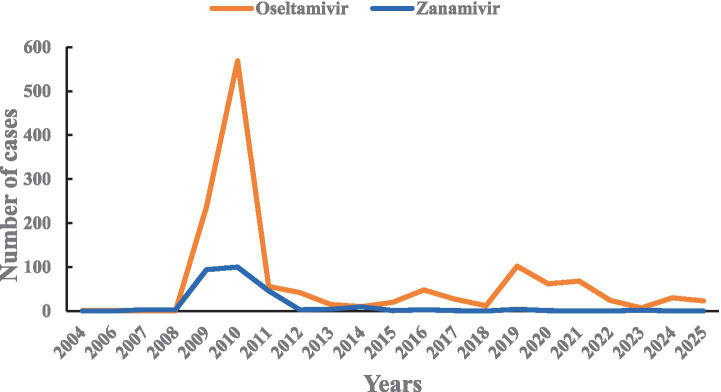
Annual distribution of neuraminidase inhibitor-associated adverse drug reactions in pregnancy.

From Q1 2004 to Q3 2025, a total of 3,687 ADE reports were retrieved in this study, including 3,211 reports for oseltamivir, 471 reports for zanamivir, and 5 reports for peramivir. The majority of reports involved pregnant women aged 18–44 years, although age information was unavailable for significant number of cases. The top 5 reporting countries were Japan (58.46%), the United States of America (14.40%), China (4.35%), the United Kingdom (2.63%), and Brazil (2.45%). The records were mainly reported by physicians (45.65%), pharmacists (21.14%), and other health professionals (17.65%), as shown in Table 2. From 2004 to 2025, the year of the highest reports of oseltamivir (569 cases) was 2010, followed by a year-on-year decline until 2019 (102 cases), when a new peak was observed. For zanamivir, 2009 (94 cases) and 2010 (100 cases) were the top 2 years with the highest number of reports, followed by a gradual decline, as shown in Figure 2.

**Table 2:** Characteristics of neuraminidase inhibitor-associated adverse drug reactions in pregnancy.

Characteristic	Report number, *n*	Report proportion, %
Age category, years		
<18	50	3.06
18–44	724	44.36
45–64	1	0.06
≥65	0	0.00
Not specified (%)	857	52.51
Medication		
Oseltamivir	3,211	87.09
Zanamivir	471	12.77
Peramivir	5	0.14
Reported countries (top 5)		
Japan	954	58.46
United States of America	235	14.40
China	71	4.35
United Kingdom	43	2.63
Brazil	40	2.45
Reporter		
Physician	745	45.65
Pharmacist	345	21.14
Other health-professional	288	17.65
Consumer	222	13.60
Not specified	31	1.90
Lawyer	1	0.06

### 3.2 Signal detection

**Figure 3:**
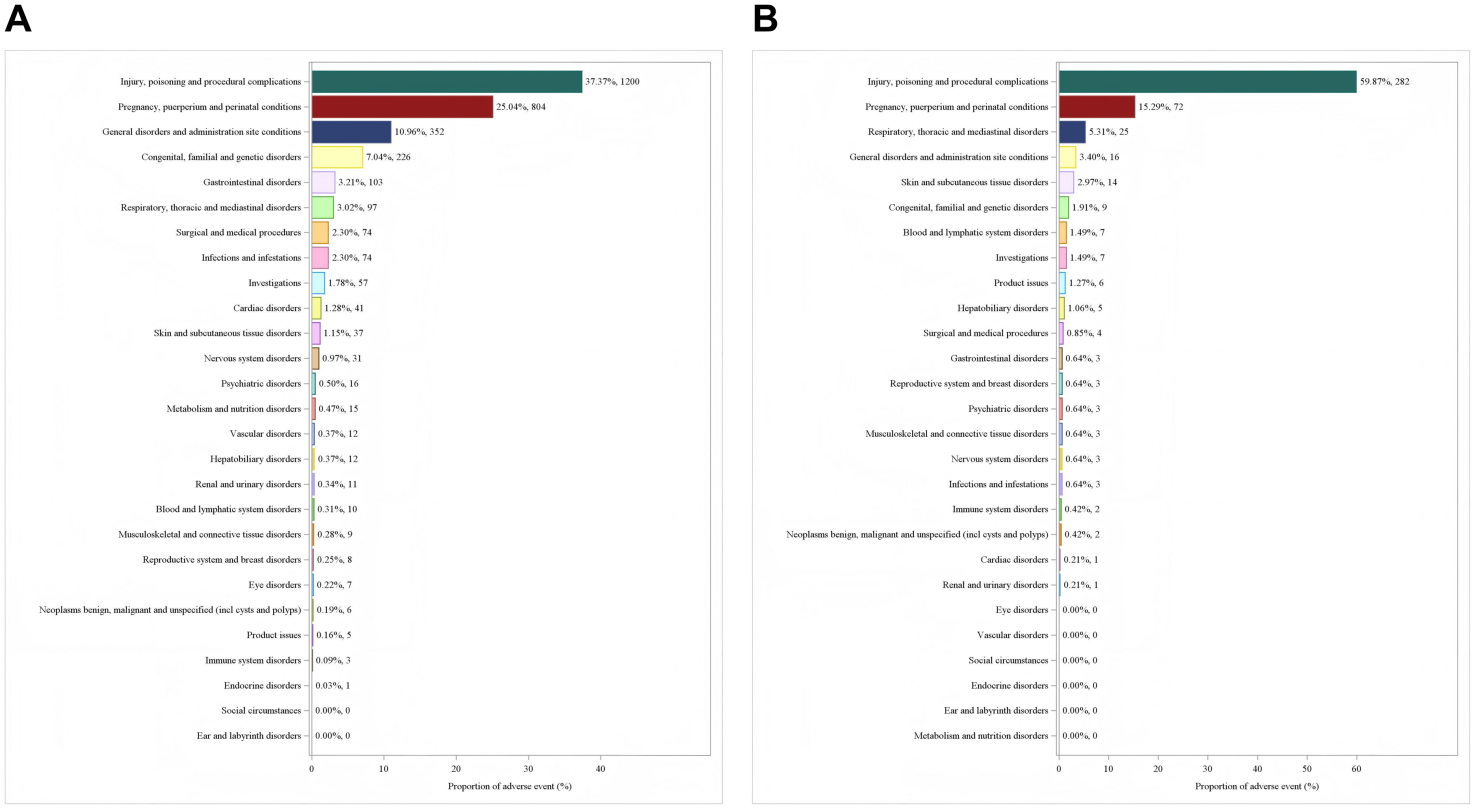
Proportion of neuraminidase inhibitor-related AEs by SOCs in pregnancy. **(A)**. Proportion of oseltamivir-related AEs by SOCs in pregnancy. **(B)**. Proportion of zanamivir-related AEs by SOCs in pregnancy. The bar plot displays the statistical distribution of neuraminidase inhibitor-related AEs across 27 SOC levels.

We compared AE signals in SOCs for neuraminidase inhibitors in pregnancy; the proportion of oseltamivir-related AEs by SOCs is shown in Figure 3A. The top five proportions of oseltamivir-related AEs by SOCs were injury, poisoning, and procedural complications (37.37%); pregnancy, puerperium, and perinatal conditions (25.04%); general disorders and administration site conditions (10.96%); congenital, familial, and genetic disorders (7.04%); and gastrointestinal disorders (3.21%). For zanamivir, the proportion of AEs by SOCs is shown in Figure 3B. The top five proportions of zanamivir-related AEs by SOCs were injury, poisoning, and procedural complications (59.87%); pregnancy, puerperium, and perinatal conditions (15.29%); respiratory, thoracic, and mediastinal disorders (5.31%); general disorders and administration site conditions (3.40%); and skin and subcutaneous tissue disorders (2.97%).

Supplementary Table S2 lists all significantly disproportionate PTs that simultaneously complied with algorithms. The top five PTs with the highest frequency of oseltamivir-related AEs were exposure during pregnancy (*n* = 681, ROR: 4.17, IC: 1.80), normal newborn (*n* = 336, ROR: 39.68, IC: 5.05), no adverse event (*n* = 284, ROR: 55.50, IC: 5.52), maternal exposure during pregnancy (*n* = 225, ROR: 1.34, IC: 0.40), and pregnancy (*n* = 119, ROR: 4.72, IC: 2.18). The top five PTs with the highest frequency of zanamivir-related AEs were exposure during pregnancy (*n* = 254, ROR 18.07, IC 3.14), delivery (*n* = 34, ROR 182.56, IC 7.33), live birth (*n* = 14, ROR 6.50, IC 2.66), maternal drugs affecting fetus (*n* = 9, ROR 3.63, IC 1.84), and overdose (*n* = 5, ROR 6.37, IC 2.66).

Ranked based on ROR, the top 30 PTs of oseltamivir-related AEs and all PTs of zanamivir-related AEs are shown in Table 3. The top five strongest PT signals for oseltamivir were proctitis (*n* = 6, ROR: 251.22, IC: 7.35), infantile acne (*n* = 4, ROR: 204.57, IC: 7.15), congenital intestinal obstruction (*n* = 3, ROR: 172.55, IC: 6.97), no adverse event (*n* = 284, ROR: 55.50, IC: 5.52), and placental necrosis (*n* = 3, ROR: 47.60, IC: 5.43). The top five strongest PT signals for zanamivir were delivery (*n* = 34, ROR: 182.56, IC: 7.33), bronchospasm (*n* = 3, ROR: 83.15, IC: 6.33), pneumothorax (*n* = 33, ROR: 19.03, IC: 4.23), exposure during pregnancy (*n* = 254, ROR: 18.07, IC: 3.14), and product quality issue (*n* = 3, ROR: 9.67, IC: 3.26).

**Table 3:** Signal strength of reports of each neuraminidase inhibitor at PT level in pregnant women.

SOC	Preferred terms	Case report	ROR (95% CI)	IC (IC-2SD)
Oseltamivir				
Gastrointestinal disorders	Proctitis	6	251.22 (92.85–679.72)	7.35 (1.47)
Skin and subcutaneous tissue disorders	Infantile acne	4	204.57 (62.97–664.64)	7.15 (0.77)
Congenital, familial, and genetic disorders	Congenital intestinal obstruction	3	172.55 (45.76–650.73)	6.97 (0.30)
General disorders and administration site conditions	No adverse event	284	55.50 (48.84–63.07)	5.52 (5.12)
Pregnancy, puerperium, and perinatal conditions	Placental necrosis	3	47.60 (14.49–156.34)	5.43 (0.37)
Pregnancy, puerperium, and perinatal conditions	Normal newborn	336	39.68 (35.30–44.60)	5.05 (4.75)
Cardiac disorders	Myocarditis	4	34.74 (12.57–96.04)	5.01 (0.80)
Infections and infestations	H1N1 influenza	4	26.68 (9.73–73.16)	4.66 (0.77)
Congenital, familial, and genetic disorders	Intestinal malrotation	7	16.79 (7.89–35.73)	4.02 (1.44)
Congenital, familial, and genetic disorders	Heterotaxia	5	16.56 (6.78–40.44)	4.00 (0.99)
General disorders and administration site conditions	Hypothermia	5	13.95 (5.73–33.99)	3.76 (0.93)
General disorders and administration site conditions	Drug effective for unapproved indication	6	13.88 (6.16–31.30)	3.75 (1.16)
Respiratory, thoracic, and mediastinal disorders	Neonatal respiratory acidosis	3	12.90 (4.09–40.66)	3.65 (0.22)
Congenital, familial, and genetic disorders	Pyloric stenosis	12	12.84 (7.23–22.82)	3.64 (1.91)
Congenital, familial, and genetic disorders	Dextrocardia	4	12.36 (4.57–33.37)	3.59 (0.59)
Respiratory, thoracic, and mediastinal disorders	Acute respiratory distress syndrome	7	11.31 (5.34–23.97)	3.47 (1.25)
Pregnancy, puerperium, and perinatal conditions	Placental infarction	4	8.81 (3.27–23.70)	3.11 (0.47)
Injury, poisoning, and procedural complications	Accidental overdose	3	8.63 (2.75–27.05)	3.08 (0.10)
Infections and infestations	Neonatal pneumonia	3	8.27 (2.64–25.90)	3.02 (0.08)
Infections and infestations	Septic shock	4	6.79 (2.53–18.24)	2.74 (0.34)
Infections and infestations	Influenza	8	6.09 (3.03–12.25)	2.59 (0.98)
Respiratory, thoracic, and mediastinal disorders	Tachypnea	8	5.97 (2.97–12.01)	2.56 (0.96)
General disorders and administration site conditions	Multiple organ dysfunction syndrome	4	5.84 (2.18–15.68)	2.53 (0.26)
Congenital, familial, and genetic disorders	Congenital pulmonary valve stenosis	5	5.43 (2.25–13.12)	2.42 (0.45)
Injury, poisoning, and procedural complications	Maternal exposure timing unspecified	34	4.81 (3.43–6.76)	2.24 (1.60)
Pregnancy, puerperium, and perinatal conditions	Pregnancy	119	4.72 (3.92–5.67)	2.18 (1.87)
Congenital, familial, and genetic disorders	Cryptorchism	9	4.35 (2.25–8.40)	2.11 (0.78)
Respiratory, thoracic, and mediastinal disorders	Hypoxia	6	4.32 (1.93–9.65)	2.10 (0.44)
Injury, poisoning, and procedural complications	Exposure during pregnancy	681	4.17 (3.83–4.54)	1.80 (1.67)
Investigations	Low Apgar score	10	3.85 (2.07–7.19)	1.93 (0.73)
Zanamivir				
Pregnancy, puerperium, and perinatal conditions	Delivery	34	182.56 (127.67–261.04)	7.33 (4.33)
Respiratory, thoracic, and mediastinal disorders	Bronchospasm	3	83.15 (26.33–262.60)	6.33 (0.47)
Respiratory, thoracic, and mediastinal disorders	Pneumothorax	3	19.03 (6.09–59.42)	4.23 (0.33)
Injury, poisoning, and procedural complications	Exposure during pregnancy	254	18.07 (15.07–21.66)	3.14 (2.87)
Product issues	Product quality issue	3	9.67 (3.10–30.13)	3.26 (0.15)
Pregnancy, puerperium, and perinatal conditions	Live birth	14	6.50 (3.81–11.06)	2.66 (1.46)
Injury, poisoning, and procedural complications	Overdose	5	6.37 (2.64–15.39)	2.66 (0.55)
General disorders and administration site conditions	No adverse event	4	4.43 (1.65–11.85)	2.14 (0.09)
Injury, poisoning, and procedural complications	Maternal drugs affecting fetus	9	3.63 (1.87–7.02)	1.84 (0.58)

## 4 Discussion

Our pharmacovigilance analysis of the FAERS database detected possible signals of ADRs in women during pregnancy associated with the use of neuraminidase inhibitors. For oseltamivir, significant AEs at SOCs were injury, poisoning, and procedural complications; pregnancy, puerperium, and perinatal conditions; general disorders and administration site conditions; and congenital, familial, and genetic disorders and gastrointestinal disorders. The five strongest AE signals were proctitis, infantile acne, congenital intestinal obstruction, no adverse event, and placental necrosis. For zanamivir, significant AEs at SOCs were injury, poisoning, and procedural complications; pregnancy, puerperium, and perinatal conditions; respiratory, thoracic, and mediastinal disorders; general disorders and administration site conditions; and skin and subcutaneous tissue disorders. The five strongest AE signals were delivery, bronchospasm, pneumothorax, exposure during pregnancy, and product quality issues. Peramivir was excluded from the disproportionality analysis due to limited data, which only had five case reports.

Pregnant women are at high risk for influenza complications. Once influenza is suspected or confirmed, antiviral treatment should be initiated as soon as possible regardless of the stage of pregnancy, without awaiting laboratory confirmation results. Therefore, neuraminidase inhibitors are widely recommended for the treatment of pregnant and postpartum women with influenza (6–8). In a meta-analysis of nine cohort studies evaluating neonatal outcomes following maternal exposure to neuraminidase inhibitors (11), the preterm birth rate, incidence of congenital malformations, and low Apgar scores were similar between the group exposed to neuraminidase inhibitors and the non-exposed infant group. Moreover, exposure to a neuraminidase inhibitor was associated with a reduced risk of low birth weight and giving birth to a small-for-gestational-age (SGA) infant. A North American cohort study comparing 112 women exposed to oseltamivir at any time during pregnancy with 604 unexposed pregnant women demonstrated similar outcomes between the two groups with respect to preterm delivery and SGA infants (20). Another Canadian study involving 55,355 pregnant women, of whom 1,237 had exposure to oseltamivir, reported no association between oseltamivir exposure and SGA, preterm birth, or low 5-min Apgar scores (21). Findings from a prospective cohort study involving 782 pregnant women based on UK national surveillance data showed no differences in the incidence of adverse pregnancy outcomes in either group compared to untreated pregnant women with influenza (22).

In our study, healthy newborn and no adverse event were among the top five PTs with the highest frequency for oseltamivir, and delivery, live birth, and no adverse event were with high frequency for zanamivir, which aligns with existing knowledge that neuraminidase inhibitors are safe and effective among pregnant women for the treatment of influenza (7, 8). However, the exposure trimester was not provided due to the lack of data; therefore, we could not evaluate the AE signals stratified by gestational period. Notably, a study provided malformation rate data stratified by gestational period (23), demonstrating that, among the 441 cases exposed during the first trimester, the incidence of congenital malformations was 2.0% (9/441), with no significant difference compared with the second-trimester exposure group (1.8%), the third-trimester exposure group (2.6%), or the unexposed control group. Therefore, oseltamivir may be considered as an antiviral treatment during pregnancy with influenza at any gestational week. Additionally, it is imperative to emphasize that influenza infection itself is an important confounding factor, and the observed adverse outcomes are likely attributable to the disease itself rather than the medications used to treat it. It has been reported that severe influenza infection and high fever are known risk factors for preterm birth and SGA (24). Therefore, the data should be interpreted more cautiously.

In our study, congenital, familial, and genetic disorders are the most frequently involved SOC ranking based on ROR for oseltamivir. AEs at the PT level of this SOC include congenital intestinal obstruction, intestinal malrotation, heterotaxia, pyloric stenosis, dextrocardia, congenital pulmonary valve stenosis, and cryptorchism. The PT of congenital intestinal obstruction was the strongest signal and has not been reported before. Therefore, further studies are needed to investigate this association. We identified intestinal malrotation as a PT of with seven cases, which has previously been described in a US case–control study (25). The study of birth defects identified a higher risk for intestinal malrotation in infants born to women exposed to oseltamivir during their pregnancy. However, given that this association was based on only three infants and the confidence interval for the surgical group was wide, the authors noted that this finding might be coincidental. Similarly, our results of data mining from the FAERS database also demonstrated the strong signal of intestinal malrotation; therefore, large sample-sized studies are needed to better confirm the association between intestinal malrotation and oseltamivir in pregnancy.

It should be noted that proctitis was the strongest signal based on ROR for oseltamivir, indicating the main adverse reactions in gastrointestinal disorders. For oseltamivir, the most frequently reported adverse reactions were gastrointestinal diseases in clinical trial results (26, 27), including nausea and vomiting. In a multicenter observational study involving high-risk outpatients treated with oseltamivir, gastrointestinal disorders were reported in 91.67% of patients experiencing adverse events, making them the most common side effects reported (28). Moreover, a disproportionality analysis of the FDA adverse event reporting system revealed that, in pediatric patients, oseltamivir was associated with 100 identified adverse event signals, of which gastrointestinal disorders ranked high among the reported issues (29). Consistent with these findings, our results suggested that gastrointestinal issues are a significant concern. It is critical as it underscores the necessity for vigilant monitoring of gastrointestinal symptoms, which could lead to complications in clinical management.

For zanamivir, bronchospasm and pneumothorax were among the top strong AE signals based on ROR, indicating a strong association with the respiratory system. As zanamivir is an inhaled antiviral medication for influenza acting directly on the respiratory tract, ADRs related to respiratory system, thoracic, and mediastinal disease systems were primarily reported in the previous studies. This confirmed the reliability of our research methodology and provided valuable insights for clinical medication. In clinical trials of zanamivir administered via inhalation, 18% of adverse events were related to the upper respiratory tract (30). Additionally, one study has shown that the trial group (zanamivir group) exhibited a greater number of patients with decreased pulmonary function compared to the placebo group (31). Another study has demonstrated that, among patients treated with oseltamivir or zanamivir for influenza, the zanamivir group exhibited a higher frequency of adverse events such as drowsiness and respiratory distress (32). The primary mechanism of zanamivir-induced respiratory adverse reactions is believed to involve direct irritation of sensitive airways on the inhaled dry powder, leading to the abnormal release of inflammatory mediators and subsequently triggering repeated contractions of bronchial smooth muscle (33). Therefore, the observed rates of bronchospasm and respiratory distress following zanamivir use suggest that healthcare providers should exercise careful scrutiny when prescribing this medication, particularly for at-risk individuals.

Our study has several limitations. First, our signal mining methods are based solely on statistical analysis using ROR and BCPNN algorithms. The ROR method exhibits more susceptibility to false-positive signals when event counts in the denominator are low. To mitigate this limitation and enhance signal specificity, we adopted a complementary analytical strategy—integrating ROR with the BCPNN method—to systematically identify and validate co-occurring ADR signals. However, both the ROR and the BCPNN methods identify only a statistical association and do not establish or imply a causal relationship (34). Second, the FAERS database is a spontaneous reporting system with varying degrees of underreporting, delayed reporting, misreporting, and incomplete information, which may lead to reporting bias in the measurement of the disproportionality report. Third, the potential confounding factors of concomitant medications—including both suspected and non-suspected agents—as well as its underlying comorbidities may influence the observed associations, necessitating additional clinical evaluation to confirm the findings. Fourth, the database only includes events caused by drug use; however, the number of total drug users is not provided; therefore, the incidence of adverse events cannot be calculated. Fifth, the notable difference in case counts across different drugs constitutes a significant methodological limitation for comparative analyses. Finally, the majority of cases examined in this study were from Japan and the United States, thereby introducing a potential geographic bias. Such a geographically concentrated sample may compromise the external validity of the findings and limit their generalizability to populations residing in other regions. Therefore, to assess the safety risks of neuraminidase inhibitors from a more accurate and comprehensive perspective, future research combining clinical trials and epidemiological studies using more rigorous prospective study designs is required.

## 5 Conclusion

This study offers crucial safety insights for antiviral treatment of influenza with neuraminidase inhibitor use among pregnant women. In the disproportionality analysis, congenital, familial, and genetic disorders are the most frequently involved SOCs for oseltamivir ranked based on ROR. The five strongest AE signals were proctitis, infantile acne, congenital intestinal obstruction, no adverse event, and placental necrosis. For zanamivir, significant AEs at SOCs were injury, poisoning, and procedural complications; pregnancy, puerperium, and perinatal conditions; and respiratory, thoracic, and mediastinal disorders; and the five strongest AE signals were delivery, bronchospasm, pneumothorax, exposure during pregnancy, and product quality issue. However, due to the limitations of the data, the causal relationship and risk level of adverse events cannot be accurately inferred. The findings of this study align with current guidelines recommending neuraminidase inhibitors as primary antiviral treatment of pregnant women with influenza and may offer valuable evidence to support the clinical use of neuraminidase inhibitors, particularly in identifying adverse events and ensuring their safe administration in pregnancy.

## Data Availability

The original contributions presented in the study are included in the article/Supplementary material, further inquiries can be directed to the corresponding author.
