# Supplementary material for: Signal mining of adverse reactions for neuraminidase inhibitors in pregnant women: a disproportionality analysis based on the FDA adverse event reporting system database
Source: Front Med (Lausanne). 2026 Jun 16;13:1821866. doi: 10.3389/fmed.2026.1821866 (PMC13315221; doi:10.3389/fmed.2026.1821866)
Supplement: Supplementary file 1 [file Supplementary_file_1.docx]

Table S1. 2×2 contingency table of disproportionality method.

| Item | Target adverse events reported | Other adverse events reported | Total |
| --- | --- | --- | --- |
| Reports with the target drug | a | b | a+b |
| All other drugs | c | d | c+d |
| Total | a+c | b+d | a+b+c+d |

Table S2. All PTs by frequency for each neuraminidase inhibitor in pregnancy women.

| **SOC** | **Preferred Terms** | **Case Report** | **ROR(95% CI)** | **IC(IC-2SD)** |
| --- | --- | --- | --- | --- |
| **Oseltamivir** |  |  |  |  |
| Injury, poisoning and procedural complications | Exposure during pregnancy | 681 | 4.17(3.83,4.54) | 1.80(1.67) |
| Pregnancy, puerperium and perinatal conditions | Normal newborn | 336 | 39.68(35.30,44.60) | 5.05(4.75) |
| General disorders and administration site conditions | No adverse event | 284 | 55.50(48.84,63.07) | 5.52(5.12) |
| Injury, poisoning and procedural complications | Maternal exposure during pregnancy | 225 | 1.34(1.17,1.54) | 0.40(0.20) |
| Pregnancy, puerperium and perinatal conditions | Pregnancy | 119 | 4.72(3.92,5.67) | 2.18(1.87) |
| Surgical and medical procedures | Caesarean section | 64 | 2.77(2.16,3.55) | 1.45(1.04) |
| Injury, poisoning and procedural complications | Off label use | 57 | 2.04(1.57,2.65) | 1.01(0.60) |
| Pregnancy, puerperium and perinatal conditions | Premature delivery | 54 | 1.89(1.45,2.48) | 0.91(0.49) |
| Injury, poisoning and procedural complications | Maternal exposure timing unspecified | 34 | 4.81(3.43,6.76) | 2.24(1.60) |
| Pregnancy, puerperium and perinatal conditions | Foetal death | 31 | 2.45(1.72,3.49) | 1.28(0.70) |
| Gastrointestinal disorders | Vomiting | 31 | 2.54(1.78,3.63) | 1.33(0.75) |
| Congenital, familial and genetic disorders | Ventricular septal defect | 23 | 2.03(1.35,3.07) | 1.01(0.36) |
| Pregnancy, puerperium and perinatal conditions | Premature labour | 19 | 1.78(1.13,2.79) | 0.82(0.12) |
| Pregnancy, puerperium and perinatal conditions | Stillbirth | 18 | 1.96(1.23,3.12) | 0.97(0.23) |
| Congenital, familial and genetic disorders | Pyloric stenosis | 12 | 12.84(7.23,22.82) | 3.64(1.91) |
| Pregnancy, puerperium and perinatal conditions | Premature rupture of membranes | 12 | 2.64(1.50,4.66) | 1.39(0.42) |
| Investigations | Apgar score low | 10 | 3.85(2.07,7.19) | 1.93(0.73) |
| Gastrointestinal disorders | Abdominal distension | 10 | 3.71(1.99,6.91) | 1.88(0.69) |
| Congenital, familial and genetic disorders | Cryptorchism | 9 | 4.35(2.25,8.40) | 2.11(0.78) |
| Infections and infestations | Influenza | 8 | 6.09(3.03,12.25) | 2.59(0.98) |
| Respiratory, thoracic and mediastinal disorders | Tachypnoea | 8 | 5.97(2.97,12.01) | 2.56(0.96) |
| Congenital, familial and genetic disorders | Intestinal malrotation | 7 | 16.79(7.89,35.73) | 4.02(1.44) |
| General disorders and administration site conditions | Death neonatal | 7 | 2.95(1.40,6.20) | 1.55(0.21) |
| Respiratory, thoracic and mediastinal disorders | Acute respiratory distress syndrome | 7 | 11.31(5.34,23.97) | 3.47(1.25) |
| Gastrointestinal disorders | Proctitis | 6 | 251.22(92.85,679.72) | 7.35(1.47) |
| Respiratory, thoracic and mediastinal disorders | Hypoxia | 6 | 4.32(1.93,9.65) | 2.10(0.44) |
| General disorders and administration site conditions | Drug effective for unapproved indication | 6 | 13.88(6.16,31.30) | 3.75(1.16) |
| Congenital, familial and genetic disorders | Heterotaxia | 5 | 16.56(6.78,40.44) | 4.00(0.99) |
| General disorders and administration site conditions | Hypothermia | 5 | 13.95(5.73,33.99) | 3.76(0.93) |
| Congenital, familial and genetic disorders | Pulmonary valve stenosis congenital | 5 | 5.43(2.25,13.12) | 2.42(0.45) |
| Respiratory, thoracic and mediastinal disorders | Neonatal asphyxia | 5 | 3.66(1.52,8.83) | 1.86(0.15) |
| Congenital, familial and genetic disorders | Dextrocardia | 4 | 12.36(4.57,33.37) | 3.59(0.59) |
| Skin and subcutaneous tissue disorders | Acne infantile | 4 | 204.57(62.97,664.64) | 7.15(0.77) |
| Infections and infestations | Septic shock | 4 | 6.79(2.53,18.24) | 2.74(0.34) |
| Cardiac disorders | Myocarditis | 4 | 34.74(12.57,96.04) | 5.01(0.80) |
| Pregnancy, puerperium and perinatal conditions | Placental infarction | 4 | 8.81(3.27,23.70) | 3.11(0.47) |
| Infections and infestations | H1N1 influenza | 4 | 26.68(9.73,73.16) | 4.66(0.77) |
| General disorders and administration site conditions | Multiple organ dysfunction syndrome | 4 | 5.84(2.18,15.68) | 2.53(0.26) |
| Congenital, familial and genetic disorders | Congenital intestinal obstruction | 3 | 172.55(45.76,650.73) | 6.97(0.30) |
| Infections and infestations | Neonatal pneumonia | 3 | 8.27(2.64,25.90) | 3.02(0.08) |
| Pregnancy, puerperium and perinatal conditions | Placental necrosis | 3 | 47.60(14.49,156.34) | 5.43(0.37) |
| Injury, poisoning and procedural complications | Accidental overdose | 3 | 8.63(2.75,27.05) | 3.08(0.10) |
| Respiratory, thoracic and mediastinal disorders | Neonatal respiratory acidosis | 3 | 12.90(4.09,40.66) | 3.65(0.22) |
| **Zanamivir** |  |  |  |  |
| Injury, poisoning and procedural complications | Exposure during pregnancy | 254 | 18.07(15.07-21.66) | 3.14(2.87) |
| Pregnancy, puerperium and perinatal conditions | Delivery | 34 | 182.56(127.67-261.04) | 7.33(4.33) |
| Pregnancy, puerperium and perinatal conditions | Live birth | 14 | 6.50(3.81-11.06) | 2.66(1.46) |
| Injury, poisoning and procedural complications | Maternal drugs affecting foetus | 9 | 3.63(1.87-7.02) | 1.84(0.58) |
| Injury, poisoning and procedural complications | Overdose | 5 | 6.37(2.64-15.39) | 2.66(0.55) |
| General disorders and administration site conditions | No adverse event | 4 | 4.43(1.65-11.85) | 2.14(0.09) |
| Product issues | Product quality issue | 3 | 9.67(3.10-30.13) | 3.26(0.15) |
| Respiratory, thoracic and mediastinal disorders | Pneumothorax | 3 | 19.03(6.09-59.42) | 4.23(0.33) |
| Respiratory, thoracic and mediastinal disorders | Bronchospasm | 3 | 83.15(26.33-262.60) | 6.33(0.47) |
